# Modeling Unobserved Heterogeneity in Susceptibility to Ambient Benzo[*a*]pyrene Concentration among Children with Allergic Asthma Using an Unsupervised Learning Algorithm

**DOI:** 10.3390/ijerph15010106

**Published:** 2018-01-10

**Authors:** Daniel Fernández, Radim J. Sram, Miroslav Dostal, Anna Pastorkova, Hans Gmuender, Hyunok Choi

**Affiliations:** 1 Research and Development Unit, Parc Sanitari Sant Joan de Déu, Fundació Sant Joan de Déu, CIBERSAM, Dr. Antoni Pujadas, 42, Sant Boi de Llobregat, 08830 Barcelona, Spain; df.martinez@pssjd.org; 2 School of Mathematics and Statistics, Victoria University of Wellington, Wellington 6140, New Zealand; 3 Department of Genetic Ecotoxicology, Institute of Experimental Medicine, Academy of Sciences of the Czech Republic, v.v.i., Vídeňská 1083, 142 20 Prague 4, Czech Republic; sram@biomed.cas.cz (R.J.S.); dostal.boleslav@gmail.com (M.D.); PastorkovaA@seznam.cz (A.P.); 4 Genedata AG, Margarethenstrasse 38, CH-4053 Basel, Switzerland; hans.gmuender@genedata.com; 5 Departments of Environmental Health Sciences, Epidemiology, and Biostatistics State University of New York at Albany School of Public Health, Rensselaer, NY 12144, USA

**Keywords:** gene-environment interaction, polycyclic aromatic hydrocarbon, asthma, single nucleotide polymorphism, air pollution

## Abstract

Current studies of gene × air pollution interaction typically seek to identify unknown heritability of common complex illnesses arising from variability in the host’s susceptibility to environmental pollutants of interest. Accordingly, a single component generalized linear models are often used to model the risk posed by an environmental exposure variable of interest in relation to a priori determined DNA variants. However, reducing the phenotypic heterogeneity may further optimize such approach, primarily represented by the modeled DNA variants. Here, we reduce phenotypic heterogeneity of asthma severity, and also identify single nucleotide polymorphisms (SNP) associated with phenotype subgroups. Specifically, we first apply an unsupervised learning algorithm method and a non-parametric regression to find a biclustering structure of children according to their allergy and asthma severity. We then identify a set of SNPs most closely correlated with each sub-group. We subsequently fit a logistic regression model for each group against the healthy controls using benzo[*a*]pyrene (B[*a*]P) as a representative airborne carcinogen. Application of such approach in a case-control data set shows that SNP clustering may help to partly explain heterogeneity in children’s asthma susceptibility in relation to ambient B[*a*]P concentration with greater efficiency.

## 1. Introduction

Asthma is complex heritable syndrome, which afflicts an estimated 300 million people worldwide [1]. A growing body of research suggests that particular subtype(s) of asthma arises from complex interactions of genetic and environmental factors during early-life prior to onset of symptoms [2,3]. Even though several environmental contributors of asthma risk are established to date, a growing body of genome-wide association studies (GWAS) has also shown substantial contributions by genomic variability [4,5]. That is, risk of asthma attributed to genetic variations has also risen in recent decades [5]. Since genetic variations of the populations are unlikely to have changed within a span of few decades, environmental exposure context-dependent increase in penetrance of the genetic susceptibility factors explain, at least partly, the underlying gene-environment interactions (GEIs) [4]. A recent review by Bønnelykke and Ober proposes examination of asthma subgroups with homogeneous phenotypes in conjunction with a thorough measurement of environmental exposures, as the starting point of GEIs investigations [4]. 

Childhood exposures to polycyclic aromatic hydrocarbons (PAHs), benzo[*a*]pyrene, a representative PAH, is a known risk factor for reduced lung function [6] symptom aggravation [7] or the onset of new symptoms [8,9]. Yet, present evidence on the mechanisms of particular subtypes of childhood asthma, namely, endotype, due to early-life exposures to PAHs remains incomplete [10]. To date, most investigations of gene-environment interaction focus on a priori-driven hypothesis of well-recognized pathways and/or established candidate genes [10]. Such approach inherently precludes the possibility of identifying novel pathways and/or candidate genes. An alternate strategy for identification of new or suspected alleles is needed for a deeper exploration of the mechanisms of PAH-associated asthma endotype(s).

Within the present investigation, we explore the feasibility of reducing dimensionality of data complexity in the host (i.e., asthma cases and healthy controls), using cluster analysis and regression trees. We identify homogeneous subgroups of the children, based on a data-driven analysis of allergy and asthma symptom severity, and identify features (e.g., SNPs) associated with each asthma severity group.

In recent decades, multiple algorithms and techniques have been developed to reduce data dimensionality, including hierarchical clustering [11,12], association analysis [13], or partition optimization methods, such as the *k*-means clustering algorithm [14,15]. Concurrent clustering of children and SNPs into homogeneous groups is known as biclustering (or block clustering or two-mode clustering, or co-clustering), in which the resulting sub-groups are expected to capture homogeneous subgroups of children. A hierarchical Bayesian procedure for biclustering [16], using mixtures has been proposed for binary data [17,18,19], for count data [20,21], and for ordinal data [22,23,24]. In addition, biclustering models based on double *k*-means are proposed in Rocci and Vichi [25] and Vichi [26]. Specifically, we re-categorize asthmatic children (along with the associated SNPs) from six to three clusters, while retaining a single category for the healthy controls. Within each of the three sub-groups, a non-parametric regression tree was fit to reduce the original 619 single nucleotide polymorphisms (SNPs) into 16 SNPs of interest, which are most robustly correlated within each of the three clusters. The initial pool of 619 candidate SNPs is chosen based on their roles in xenobiotic metabolism, detoxification, induction and/or repair of oxidative damage, initiation and/or enhancement of the inflammatory immune responses and DNA repair from exposure to air pollution [27,28,29,30].

Subsequently, we posit that while the individual SNP may pose very small effect individually, they jointly account for a significant proportion of variation in the children’s susceptibility to asthma per unit exposure to ambient B[*a*]P, as proposed by polygenic inheritance [31]. We test whether the variant genotypes *en masse*, associated with given sub-group, contribute to different odds of asthma diagnosis per same unit increase in ambient B[*a*]P concentration. We compare the odds of asthma per unit increase in the ambient concentration B[*a*]P and after calculating polygenic scores for each subgroup.

## 2. Materials and Methods

### 2.1. Data

#### 2.1.1. Study Sites

Equal number of the cases and the controls were enrolled from the city with historically high air pollution level (i.e., Ostrava) as well as five rural background sites (i.e., Southern Bohemian towns), in the Czech Republic [32]. Ostrava (i.e., the high exposure site), near the Polish border, has maintained a high concentration of coal mining, coal processing, and metallurgical industries since the 18th century [33]. Among the four districts within Ostrava, the cases and the controls were enrolled from the most polluted district (i.e., Radvanice–Bartovice), with a highest district mean for B[*a*]P during November 2008 (11.4 ng/m^3^), compared to the City’s annual mean value (9.3 ng/m^3^) [34]. In contrast, the rural background region (with ~24,000 populations) demonstrated mean B[*a*]P concentration of 2.5 ng/m^3^ during the same period [32]. Within the rural site, predominant local air pollution sources include indoor heating and vehicle exhaust emissions [33]. In contrast, the urban sites are characterized by industrial sources, in addition to the residential and vehicular ones. The ambient air pollution effect on the children’s gene expression levels [32], the micronuclei frequency [35], proteins and lipids peroxidation have been published [36].

#### 2.1.2. Case and Control Children

Case and control definition have been published [37]. Briefly, all children were given lung function, bronchodilation, and skin prick tests by an allergist, if he/she were flagged by a primary care physician as a possible/probable case. Asthma case definition were based on the positive results for the following criteria. (1) Child has a positive diagnosis by an allergist of ‘current’ asthma using *International Classification of Diseases* (ICD), *Tenth Revision* [38] codes within their medical record. (2) The child currently receives asthma medication. (3) The child has a clinical lung impairment based on spirometry test within the past 12 months. (4) The child was positively responsive to a bronchodilatation test during the last 12 months. (5) In addition, all children were given allergy skin tests (Phadiatop, Pharmacia & Upjohn Diagnostics, Uppsala, Sweden) for airborne allergens. Diagnostic reliability of ICD-10 diagnoses in physician visits or hospitalizations have been demonstrated in our earlier investigations [39]. In contrast, the control children were defined as those who were free from any of the above conditions. Each case was matched to a control according to the enrollment site, age group, and gender. In addition, all parents filled out a questionnaire on the children’s medical history and life-style choices. The questionnaire included questions on the children’s birth weight, length of gestation, duration of breastfeeding period for the first six months of life, body weight and height, allergy, positive history of asthma episode, allergic rhinitis episode, atopic dermatitis episode within the past 12 months, any antibiotic use within the two weeks of sampling, parental smoking history. The ethical committee of the Institute of Experimental Medicine, Academy of Sciences of the Czech Republic, approved the study. The parents of the children signed an informed consent, according to the Helsinki II declaration.

#### 2.1.3. Air Pollution Monitoring of Polycyclic Aromatic Hydrocarbons

PAHs are measured using a Versatile Air Pollution Sampler (VAPS) [40]. Particle-bound PAHs are extracted from filters and a quantitative chemical analysis of PAHs was performed by high-performance liquid chromatography (HPLC) with fluorescence detection according to the US Environmental Protection Agency (EPA) method [41]. The mean daily level is measured once every three days for total of 10 days/month in the background region; once every six days for total of 5 days/month in the high exposure region. The difference in measurement frequency was determined by the local government, according to the availability of the funds, public’s demand, and scientific opinion of the Czech Hydrometeorological Agency. Details regarding quality assurance and control have been described [39,42,43].

#### 2.1.4. DNA Extraction

Children’s sputum sample was incubated at 50 °C in a water bath for a minimum of 1 h followed by incubation with ORAgene Purifier on ice for 10 min (DNA Genotek, Ottawa, ON, Canada). DNA was precipitated with 95% ethanol, and then diluted in TE buffer (10 mM Tris, 0.1 mM EDTA, pH 8.0). DNA concentrations were measured between 50 and 80 ng/μL using a NanoDrop 1000 Spectrophotometer (Thermo Fischer Scientific, Wilmington, DE, USA).

#### 2.1.5. SNP Detection

Quantification of SNP on Glutathione *S*-transferase Mu 1 (*GSTM1*) Glutathione *S*-transferase theta-1 (*GSTT1*), plus 95 genes have been described [42,43]. The SNPs from the 95 genes of interest were selected based on their known role of protection against oxidative injury, metabolism of xenobiotics, DNA repair, and/or immune and inflammatory responses. The SNPs of interest were chosen from SNP500 Cancer Database (http://snp500cancer.nci.nih.gov/). Only the SNP with the minor allele frequency >5% were included. Custom-designed 96-sample panels were employed to detect 768 SNPs using GoldenGate genotyping assay on a BeadStation 500GX system (Illumina, San Diego, CA, USA) according to the manufacturer’s instructions. As a quality control measure, 12% of the DNA samples were randomly genotyped, yielding 100% concordance. We removed the samples and SNPs with p10 GC score <0.40 and/or call frequency score <0.60 for all SNPs from further analysis. Furthermore, polymorphisms with poor clustering quality (149 SNPs) were removed, yielding 619 SNPs for further analysis.

#### 2.1.6. Cotinine and Vitamin Assays

Urine sample was used to validate self-reported tobacco exposure. Urinary cotinine levels were analyzed by radioimmunoassay [35]. Cotinine >450 ng/mg of creatinine was considered a cut-off value for active smoking status; 20–449 ng/mg was deemed cut-off for passive smoking status. Blood sample (~40 mL) was drawn by venipuncture into vacuettes containing lithium heparin (for the vitamin C assay) or EDTA (for the vitamin A, E assays). All tissue samples were stored at 4 °C and transported within 18 h of collection to the Department of Genetic Ecotoxicology for Vitamin A, C, and E analyses [44,45].

#### 2.1.7. Allergy and Asthma Severity Index

We recoded the dichotomous asthma outcome, into an ordinal variable, allergy and asthma severity index (AASI) (see Table 1). The asthmatic children (yes/no) were subdivided, according to the age at onset of atopic dermatitis, allergic sensitization, wheezing symptoms, as well as the results of the spirometry tests as proxy for the severity of current asthma as shown below [46,47].

A Jonckheere–Terpestra (JT) test was used to assess a linear trend, without assuming an underlying normal distribution of age at *α* = 0.05.

### 2.2. Biostatistical Methods

The variable ambient concentration B[*a*]P was transformed to a natural log scale as it was right-skewed. The children’s self-reported cigarette smoke exposure was validated with urinary cotinine concentration. The secondhand smoking status was defined as creatinine-adjusted cotinine 20 to 449 ng/mg; and current active smoking status as cotinine ≥450 ng/mg [48]. In addition, parental report of the number of smokers at home was correlated with the child’s creatinine-adjusted cotinine level, using Spearman’s non-parametric ranked agreement. All active smokers (7% of asthmatics and 6% of controls) were removed from further analysis. The distributions of individual SNPs were checked for Hardy-Weinberg equilibrium (HWE) using a chi-square test.

#### 2.2.1. Exposure Window of Interest

Ambient B[*a*]P concentration at a monitor nearest to each child’s home was matched. Consistent with our earlier analysis [39], optimal exposure window was defined as average 30-day periods. However, as the childhood susceptibility to asthma exacerbation is expected to be dependent on the age of the child, we compared the representativeness of the period of interest (i.e., 30-day average for November 2008) against November 2007, and November 2006, respectively. Routine B[*a*]P monitoring program began in 2006 for Ostrava [49].

#### 2.2.2. Identification of Confounders

Same set of putative confounding of B[*a*]P-asthma association by following variables were examined, including age, gender, total number of smokers in the family, body mass index, plasma levels of Vitamin C (mg/L), Vitamin A (mg/L), and Vitamin E (mg/L), season of delivery (indicator variables, fall, winter, and spring), and gestational age at delivery (see Table 2). That is, univariate association between each of above variable with ambient B[*a*]P and the asthma and allergy diagnosis, respectively, were examined through linear correlation coefficients as well as Pearson χ^2^ test.

#### 2.2.3. Multivariate Analyses

In the logistic regression model, we retained the variable as a possible confounder in the model if the variable induced >10% change in the regression coefficients of B[*a*]P. The largest initial logistic regression model included following predictors: age, gender, total number of smokers in the family, obesity (body mass index ≥30), plasma levels of Vitamin C (mg/L), Vitamin A (mg/L), and Vitamin E (mg/L), season of delivery (indicator variables, fall, winter, and spring), birthweight and gestational age at delivery. The final model adjusted for gender, age, obesity, and total number of smokers at home. Regression diagnostics to determine the robustness of the estimated odds ratios were performed by removing the influential values, which were defined as a value 3-times greater than 75th percentile.

#### 2.2.4. Double *k*-Means

This technique extends well-known vector quantification of *k*-means methodology [50]. Double *k*-means method [25,26] groups the individuals (e.g., children) and the features (e.g., SNPs) simultaneously. The model identifies clustering structures by numerical solution of a least-squares algorithm and highlights dependence between children and SNPs. We tried a finite number of cluster for both children and SNPs and the criterion to decide the optimal number of clusters is obtained as a trade-off between the sum of squares between cluster (SSB) and within clusters (SSW) for all possible number of clusters tested. The optimal number of clusters of children and SNPs was three, which can be summarized as: healthy control children (Cluster 1), mild-moderate case children (Cluster 2), and severe cases (Cluster 3).

#### 2.2.5. Regression Trees

Environmental health analyses often involve modeling the relationship between a response (e.g., health outcomes) and a set of explanatory variables for the purposes of quantifying the exposure—health outcome associations, describing patterns and processes, or making spatial or temporal predictions. The regression tree [51] is a non-parametric model, which is suitable for data with sample size >100. However, the application of regression trees requires the imposition of fewer model assumptions than their traditional parametric counterparts. Thus, regression trees can select from among many predictors those and their possible complex interactions that are most important in determining the outcome variable to be explained. This is an advantage for our data set because the number of predictors is high, and the modeling of all possible interactions makes the parametric counterparts infeasible. We apply this regression to each of the clusters obtained with the double *k*-means approach to select the primary SNPs and their possible interactions. The optimal tree maximally reduces deviance and it will indicate the number of final set of SNPs, as the structure of regression tree.

#### 2.2.6. Re-Categorizing Ordinal Outcome

An ordinal outcome variable is one with a categorical data scale, which describes order. Each ordinal level refers to a greater or smaller magnitude of a certain characteristic than another level. For ordinal outcomes, there is a clear ordering of the levels, but the absolute distances among them are unknown. Thus, the degree of dissimilarity among the adjacent levels of the scale in an ordinal variable might not necessarily be always the same. For instance, the difference in the severity of an injury expressed by level 2 rather than level 1 might be much more than the difference expressed by a rating of level 10 rather than 9. In addition, the utilization of the first q positive integers as labels does not imply that there is an equal space among ordinal categories. We used the ordered stereotype model in our analysis to help us to decide the correct number of categories and, also, to determine the spacing among categories. This model was proposed by Anderson (1984) [52] and is a paired-category logit model as nested between the adjacent-categories logit model and the standard baseline-category logit model. One of the main advantages of using an ordered stereotype model to fit ordinal outcome is that it is able to determine a new spacing among ordinal levels, which is dictated by the data. This is information no evaluated with other common ordinal regression models such as the proportional odds model or the adjacent-categories logit model.

#### 2.2.7. Polygenic Risk Score (PRS) Calculation

PRS is typically estimated as a weighted sum of the number of risk alleles. However, a priori weight information (i.e., published ORs of asthma) are not available for most of the 619 SNPs. Therefore, we used equal weight for all high-risk alleles. The odds of asthma diagnosis per high B[*a*]P exposure was compared across the sub-groups.

#### 2.2.8. Statistical Software

All analyses were performed with the statistical package R 3.2.3 (R Foundation for Statistical Computing. Vienna, Austria) and SPSS version 20.0.1 for Windows (SPSS Inc., Chicago, IL, USA).

## 3. Results

### 3.1. Re-Categorizing Ordinal Outcomes

The fitting of a new spacing among ordinal levels allows that two ordinal adjacent levels with “close” values can be collapsed. Initially, asthma severity was defined as a 7-level uniform and equidistant ordinal variable (Table 2). After fitting an ordered stereotype model to this variable, the fitted spacing dictated by the data is as shown in Table 2 row (a). The original equally spaced 7-level scale (1–7) is transformed into an estimated non-equally spaced 7-level scale whose values are (1, 4.954, 6.784, 6.988, 6.994, 6.994, 7). The last four categories were indistinguishable, and thus, could be merged. The final 4-level categories after collapsing those categories are highlighted in boldface in Table 2, rows (b,c).

As shown in Table 2 row (a), the scores of the last four categories are very close to each other (from 6.988 to 7). Accordingly, the categories 4–7 were re-categorized into one, thereby yielding a 4-level ordinal variable (Table 2, rows (b,c)). The results in Table 2 row (c) still assume equal spacing between ordinal categories. We replaced the original ordinal response values with the non-equal, yet continuous spacing obtained from fitting the ordered stereotype model (Table 2, row (c)). We used this final adjustment of the number of categories in the asthma severity variable to apply the double *k*-means and, subsequently, the non-parametric regression trees. There are two main advantages of collapsing adjacent levels in an ordinal variable: (a) to avoid ordinal levels that are not distinguishable in terms of predictive power, and (b) to reduce the cost of collecting the same type of data in future similar studies.

### 3.2. Double k-Means

**Figure 1:**
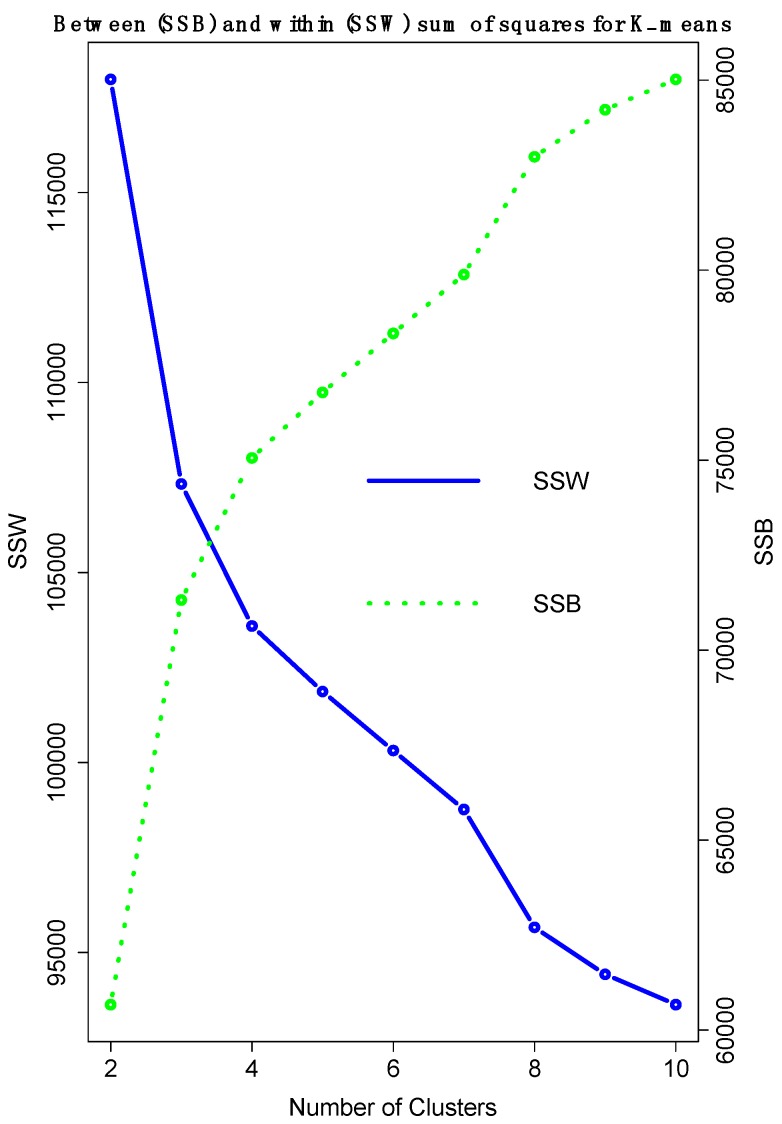
Sum of squares between cluster (SSB) and within clusters (SSW) for *k* = 2,…, 10 number of clusters for the children’s asthma data set.

Based on the double *k*-means technique, sets of SNPs most closely associated with each severity level are identified. We considered from *k* = 2 to *k* = 10 groups of SNPs and children taking 10 random starting points and we used the recategorized ordinal scale of the asthma severity. The goal of SNP clustering was to identify a set with smallest within-group variability and largest between-group variability. Figure 1 displays the evolution of the sum of squares between cluster (SSB) and within clusters (SSW) for all possible number of clusters tested.

The best trade-off between SSB and SSW is observed when *k* = 3 (SSB < SSW) or *k* = 4 (SSB > SSW). Observing the resulting clustering structure according to asthma severity, the best solution is 3 clusters of children and SNPs. Thus, the children within the *k* = 3 cluster are very similar, but very different from the children of the other clusters. Cluster 1 contains only the healthy control children (severity = 1); Cluster 2 contains mild-moderate case children with severity = 2.977 (i.e., the level 2 in the 4-level scale; Table 2, row (b)); and Cluster 3 includes the most severe cases (i.e., the levels 3 and 4 in the 4-level scale; Table 2, row (b)).

### 3.3. Demographic Traits of the Clusters of Children

Summary statistics of the three clusters (cluster 1-Reference group, cluster 2-Mild/moderate, and cluster 3-Severe outcome) are shown in Table 3. The severe asthma cases (cluster 3) had their first clinical diagnosis by 2 years of age, compared to mean first diagnosis age of 6 years among the controls (*p* < 0.001). Similarly, the cluster 3 was also associated with a longest history of corticoid treatment, compared to the controls (*p* < 0.001). Cluster 2 and 3, were associated with also respectively associated with significantly higher mean ambient B[*a*]P concentration (*p* < 0.001). At the same time, the cluster 3 was not associated with significantly elevated urinary cotinine concentration (*p* = 0.329), or a higher number of cigarette smokers at home (*p* = 0.108). Significantly lower proportion of mild/moderate (cluster 2) and severe (cluster 3) cases were enrolled from the rural background site, compared to the controls (*p* = 0.001).

### 3.4. Regression Trees Using ln(B[a]P)

A non-parametric regression tree models were fit for each cluster, in which the response variable was the logarithm of benzo[*a*]pyrene, ln(B[*a*]P), and the predictors were the resultant SNPs from the cluster analysis. Using ln(B[*a*]P) >2, which is the cut-off for selecting the levels of the tree having a statistically significant change in deviance, the significant SNPs within each cluster are shown in Table 4.

Table 4 summarizes biclustering ID of the original AASI categories, count of children in each category, and SNPs associated with each biclustering ID. A total of 16 SNPs were identified. While mild/moderate outcome (cluster 2) group was associated with five SNPs, the severe outcome group (cluster 3) was associated 11 SNPs, one of which have been identified in our earlier investigation (rs2070673 in gene *CYP2E1-07*) [37].

### 3.5. Application of Bi-Clustering Methods to Estimation of ln(B[a]P) Association with Asthma

**Figure 2:**
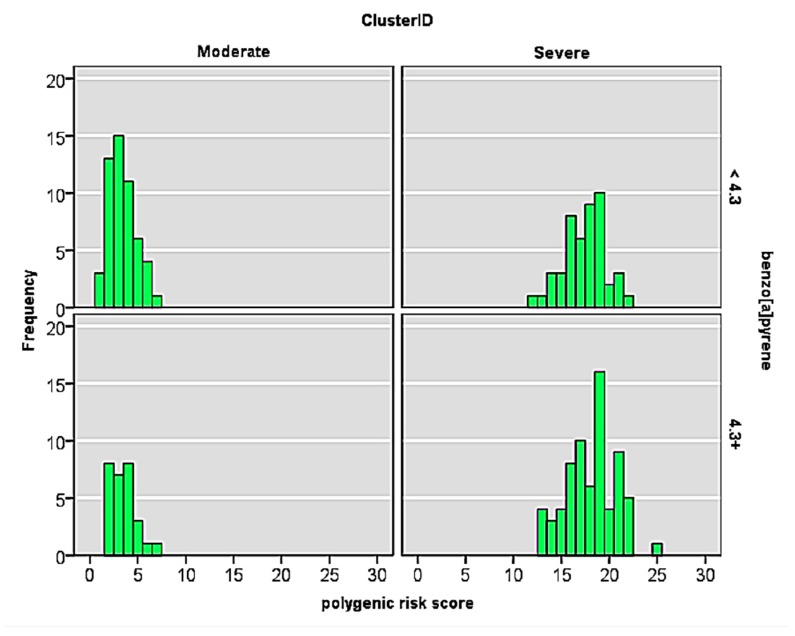
Distribution of polygenic risk score for moderate-(cluster 2) and severe outcome (cluster 3) groups of children.

As shown in Figure 2, mild/moderate outcome group was associated with an overall lower polygenic risk score (3 ± 1, mean ± SD), compared to the children in severe outcome cluster (mean polygenic risk score, 18 ± 2).

Table 5 summarizes the distribution of the polygenic risk scores for the children in moderate and severe outcome groups, respectively.

In addition, we compared the demographic traits of the children within a particular cluster (see please Table 6), according to their polygenic risk score. There was no indication of trend in demographic, disease history, and/or exposure history according to the polygenic risk scores within the respective clusters (all *p*-values > 0.05).

As shown in Table 7, the adjusted odds of the asthma outcome per (ln) unit increase in ambient B[*a*]P concentration were overall similar between moderate-risk (aOR, 2.4; 95% CI, 1.0–5.4) and the severe outcome groups of children (aOR, 2.7; 95% CI, 0.8–9.3). However, following further stratification according to the polygenic risk scores, the same unit increase in ambient B[*a*]P concentration was associated with somewhat increased adjusted odds of the asthma (aOR, 3.8; 95% CI, 0.5–31.5) outcome for both moderate- and severe outcome (aOR, 5.2; 95% CI, 0.8–36.1), compared to the low polygenic score groups (Table 7).

## 4. Discussion

Allergic asthma, allergic rhinitis, and atopic dermatitis represent the most burdensome childhood diseases in the world [53]. In the US, an estimated 40 million (or 13%) people suffer from asthma during their lifetime. To date, clinical and public health interventions to mitigate symptom exacerbations have been met with limited success, because the existing strategies do not address possible heterogeneous pathogenesis of multiple asthmas with apparently common symptoms. In spite of growing evidence that asthma is a heterogeneous collection of diseases, most investigations to date have ignored the complex contributions by genetic, environmental, dietary, and/or social domains [54,55]. As of today, the predominant research approach targets single risk domain (e.g., “asthma genes”) for an identification of singular and potent causal factors. Thus, there is an urgent need to integrate age-sensitive multi-axial functional genomic data, in order to clarify how early-life exposures to air pollution, and c-PAHs in particular, brings about clinical and preclinical symptoms (i.e., phenotype) via specific molecular mechanisms (i.e., endotype).

This paper introduces a two-step technique to reduce the heterogeneity of the host traits, through clustering of single nucleotide polymorphisms (SNPs) associated with the symptoms. According to our methodology, the analysis suggests for the first time that the children with high polygenic risk score have markedly higher odds of asthma outcome per unit increase in ambient B[*a*]P concentration, within each severity outcome group (i.e., biclustering ID). In a first step of the analysis, we apply an unsupervised learning algorithm method (double *k*-means) and non-parametric regression trees to find a biclustering structure of children according to asthma severity and SNPs where the dimensionality of the SNPs is reduced. In the second step, we apply conditional logistic regression for each polygenic risk score group, nested within severity groups, against the healthy controls using B[*a*]P as a representative airborne carcinogen.

*k*-Means algorithm creates a partition of clusters of the data space, where each observation belongs to the cluster with the nearest mean. It allows an efficient implementation for obtaining clusters, which are easy to interpret. The procedure yield computationally expedient results, and is available in most of the statistical software. We implement the double *k*-means algorithm version in the statistical package R 3.2.3. Unfortunately, there is not a general and well-established methodology to decide the unknown number of groups hidden in the data set. The criterion used in this article is a trade-off between the sum of squares between and within clusters. Although it is not the case for the data set analyzed here, one of the drawbacks of this technique is that it might be sensitive to outliers.

Regression trees [51,56] are commonly used in data analysis with the objective of creating a model, which is robust and flexible, easy to interpret, and predicts the value of an outcome based on the values of several predictors. When the sample size like is large as the data set used here the use of this technique has advantages such as latent nonlinear relationships between predictors do not affect the performance of the tree, is easy to interpret, and performs variable selection, which is our interest in this paper. One of the limitations of using this non-parametric tool is the lack of a standardized validation test to assess the goodness-of-fit of the model. One option would be to use cross-validation to obtain an R-square, which might be used to compare with their equivalent parametric counterparties. Additionally, we chose to use non-parametric regression tree models to do variable selection because it is easy to replicate by researchers and practitioners in the field as it is implemented in most of the commonly used statistical software. There are other approaches that could be used to provide variable selection such as Least Absolute Shrinkage and Selection Operator (LASSO), Gibbs Variable Selection (GVS), and Stochastic Search Variable Selection (SSVS).

Childhood exposures to polycyclic aromatic hydrocarbons (PAHs), are associated with allergic sensitization and early-onset wheezing symptoms in children [8,9], and acute aggravation of existing asthma [57]. However, the sources of variability in host susceptibility to PAH remain unclear [57]. Here, extreme seasonal variations in ambient PAH concentrations contribute to overall homogeneous distribution of ambient PAH concentration within relatively confined geographic locations of interest [34]. For example, mean for B[*a*]P concentration in the polluted urban location during our study period (November, 2008) was 11.4 ng/m^3^, approximately 5-times higher than that in background location (2.5 ng/m^3^) during the same period [32]. Since the 75th percentile of B[*a*]P is <0.5 ng/m^3^ in both New York City, US [58], and London, UK [59], distinct spatiotemporal B[*a*]P contrasts in Czech Republic allow us to detect the health effects using smaller sample size, than that typically required for G × E studies [58,60,61,62,63].

PAHs represent the most potent genotoxic proportion of the inhaled PM_2.5_, and PM_10_ [64]. Our earlier work has shown that B[*a*]P concentration >1.0 ng/m^3^ could induce DNA damage, oxidative damage [65], genomic translocations [66], micronuclei [36] and DNA fragmentation in sperm [67]. Prenatal exposure to ambient air polluted by PAHs is associated with a heightened risk of intrauterine growth restriction (IUGR) [58,60,68], preterm delivery [58], reduced neonatal height and gestational age [69]. PAH mixture represents an environmental risk factor for not only swift aggravation of asthma [57], but also an early-life induction [9]. However, the evidence gathered to date is either model-based or indirect in terms of the human exposure estimation [57]. For example, in vitro studies of human cells have demonstrated that B[*a*]P suppresses both humoral (B-cell mediated) and cellular (T cell-mediated) immune responses [70]. B[*a*]P also impairs aryl hydrocarbon receptor (AhR)-regulated signaling pathways [70]. Using human macrophages exposed to B[*a*]P, gene expression analysis indicated that biological functions linked to immunity, inflammation and cell death were most severely affected, including AhR-mediated p53 pathways [71].

Strengths of the present investigation include direct quantification of ambient B[*a*]P levels. As all children within the both of our target regions are served by the primary care clinicians within our study, our sample captures population-representative group of children. Furthermore, even though our study design is cross-sectional, and therefore enrolled existing asthma cases, this is unlikely to have influenced the identification of high-risk SNPs. That is, high-risk SNPs are unlikely to have been influenced by asthma severity. Additionally, assigning scores to ordinal categories in the asthma severity response gives an easy way to analyze the data. Future examination of assigning clinical basis of asthma symptom severity is warranted for creation of ordered categories. By using the ordinal scoring scheme of asthma outcome, ordinary linear models could be applied. However, if there were little clinical or biological rationale for the spacing between adjacent categories, the use of an ordered stereotype model is expected to yield more optimal results. This is because the ordered stereotype model does not assume equally spaced distance across the ordinal outcomes. The estimation of the spacing among ordinal responses is an improvement over other ordinal data models, such as the proportional odds model and continuation-ratio model. Finally, by analyzing the data set, we found several clusters and therefore show the presence of unobserved heterogeneity, which is most likely to exist in almost all this type of data sets. Therefore, the two-step technique presented in this article can assist practitioners in partly reducing heterogeneity in children’s asthma phenotypes in relation to ambient B[*a*]P concentration.

At the same time, several limitations are noted. First, our sample size is modest. The associated 95% confidence interval of the (ln) unit odds of asthma was wide, likely due to the limited sample size. Furthermore, PAH has been shown to have high mutual correlations with other air pollutants. Therefore, residual confounding by other correlates of B[*a*]P in air (e.g., metals) could not be ruled out. Future analysis should account for the effects of metal components within PM2.5 [37]. In addition, another limitation is that double *k*-means could fail to find a correct clustering structure if the number of local minima in the data set is large. A good option to avoid local maximum is trying multiple well-separated starting points. As a possible extension of our work, we think that a comprehensive simulation study could be set up to compare our approach against other polygenic risk score approaches such as PRSice, PredictABEL, and gtx. The reproducibility of the polygenic score, based on our identified SNPs, as an efficient predictor of asthma severity requires validation and it would be another future direction to take.

## 5. Conclusions

Our approach demonstrates an efficient strategy to reduce host heterogeneity in underlying susceptibility. Validation of our observation through future prospective cohort study design is warranted.

## Figures and Tables

**Table 1:** Recoding the dichotomous asthma outcome, into an ordinal variable, allergy and asthma severity index (AASI).

AASI	Clinical Diagnoses	Age at Onset
1	none	
2	allergen sensitized	any age
3	allergen sensitized; atopic dermatitis	At least one at ≤24 months
4	allergen sensitized; atopic dermatitis; wheezing	At least one at ≤24 months
5	allergen sensitized; atopic dermatitis; wheezing; positive bronchodilation result	At least one at ≤24 months
6	allergen sensitized; atopic dermatitis; wheezing; positive bronchodilation result; and upper respiratory infection	At least one at ≤24 months
7	allergen sensitized; atopic dermatitis; wheezing; positive bronchodilation result; upper respiratory infection; and rhinitis	At least one at ≤24 months

**Table 2:** Re-categorization of the ordinal variable asthma severity variable. The original equally spaced 7-level scale was estimated and transformed into a new non-equally spaced 7-level scale (highlighted in italic in row (a)). The final 4-level categories after collapsing their categories are highlighted in boldface in rows (b) and (c).

Recoding	Category of Asthma Severity	1	2	3	4	5	6	7
(a)	Original	*1*	*4.954*	*6.784*	*6.988*	*6.994*	*6.994*	*7*
	Frequency	179	81	76	33	12	12	1
(b)	Intermediate	**1**		**2**		**3**		**4**
	Frequency	179		81		76		58
(c)	Rescaled	**1**		**2.977**		**3.892**		**4**
	Frequency	179		81		76		58

**Table 3:** Demographic traits of the three cluster groups: cluster 1-Reference group, cluster 2-Mild/moderate, and cluster 3-Severe outcome. The values are rounded to the nearest unit.

Demographic Traits	Reference Group (*n* = 179)				Mild/Moderate (*n* = 81)				Severe Outcome (*n* = 124)				*p*
	Mean	SE	Min	Max	Mean	SE	Min	Max	Mean	SE	Min	Max	
Child’s age	11	0	6	16	12	0	6	16	11	0	6	16	0.201
Age at first clinical diagnosis (months)	82	32	35	143	72	5	24	165	24	4	0	171	<0.001
Corticoid treatment (months)	0	0	0	0	31	5	0	137	33	3	0	120	<0.001
B[*a*]p (ng/m^3^)	4.4	0.2	0.5	11.2	6.7	0.8	0.5	28.0	8.8	0.7	0.5	28.0	<0.001
No. smokers at home	1	0	0	6	1	0	0	4	1	0	0	8	0.312
BMI (kg/m^2^)	18.18	0.25	6.5	30.5	18.89	0.37	13.6	29.7	19.09	0.37	13.7	38.5	0.075
Gestational age (weeks)	40	0	32	42	40	0	33	42	39	0	26	42	0.085
Vitamin A (mg/L)	0.7	0.0	0.2	4.2	0.7	0.0	0.1	1.8	0.7	0.0	0.2	2.7	0.908
Vitamin C (mg/L)	7.3	0.3	1.3	16.0	7.049	0.4	2.2	16.4	6.9	0.3	1.9	13.4	0.651
Vitamin E (mg/L)	10.1	0.3	2.8	21.5	10.1	0.4	2.8	22.7	10.7	0.4	3.2	23.0	0.346
Enrollment site (rural)	83	43%			56	29%			55	28%			0.001
Gender (female)	85	48%			33	41%			48	39%			0.278
Mother smoker (yes)	45	25%			25	31%			32	26%			0.609
Father smoker (yes)	83	46%			34	42%			48	39%			0.440

**Table 4:** Clustering structure for children’s asthma data set.

Biclustering ID	AASI	*n*	%	Gene Name	SNP	Native/Variant Allele
Reference group (i.e., 1)	0	179	100%		n.a.	
Mild/Moderate	1	81	100%	*ERCC4-42*	rs744154	C/G
(i.e., 2.98)				*MBL2-38*	rs1031101	A/G
				*XRCC3-04*	rs1799796	T/C
				*GSTT1*		+/−
				*GSTP1*	rs1695	A/G
Severe outcome	2	76	61%	*IL6-06*	rs2069832	A/G
(i.e., 3.93)	3	33	27%	*CYP1B1-82*	rs151257	T/G
	4	12	10%	*GSTM3-01*	rs7483	A/G
	5	2	2%	*NQO1-01*	rs1800566	A/G
	6	1	1%	*IL1RN-04*	rs380092	A/T
				*CD14-06*	rs4914	C/G
				*CYP2E1-07*	rs2070673	A/T
				*LIG1-03*	rs20579	A/G
				*GATA3-46*	rs925847	A/G
				*IL1A-02*	rs1800587	A/G
				*CYP1B1-42*	rs162557	A/G

**Table 5:** Polygenic risk scores for moderate- and severe outcome clusters.

Mild/Moderate (Cluster 2)			Severe Outcome (Cluster 3)		
Polygenic Risk Score	*n*	%	Polygenic Risk Score	*n*	%
1–2	24	30%	12–16	35	30%
3	22	27%	17–18	31	27%
4	19	24%	19–20	32	27%
5–7	16	20%	21–25	19	16%

**Table 6:** Demographic traits of the clusters.

Biclustering ID	Polygenic Risk Score Categories																*p*
Mild/Moderate (Cluster 2)	PRS ≤ 2 (*n* = 24)				PRS 3 (*n* = 22)				PRS 4–5 (*n* = 19)				PRS ≥ 6 (*n* = 16)				
	Mean	SE	Min	Max	Mean	SE	Min	Max	Mean	SE	Min	Max	Mean	SE	Min	Max	
Child’s age	12	1	6	16	12	1	6	16	11	1	6	15	12	1	8	16	0.751
Age at first clinical diagnosis (months)	73	7	25	155	72	8	24	165	63	8	28	163	70	10	24	160	0.831
Corticoid treatment (months)	24	6	0	103	39	7	0	102	26	7	0	108	30	11	0	137	0.492
No. smokers	1	0	0	3	1	0	0	2	1	0	0	2	1	0	0	4	0.083
BMI (kg/m^2^)	19	1	14	30	19	1	14	27	18	1	14	23	19	1	16	25	0.824
Vitamin A (mg/L)	1	0	0	1	1	0	0	2	1	0	0	1	1	0	0	1	0.353
Vitamin C (mg/L)	7	1	3	14	7	1	2	12	7	1	4	16	7	1	4	14	0.930
Vitamin E (mg/L)	10	1	3	15	10	1	5	17	9	1	4	19	11	1	6	23	0.479
Urban site (*n*, %)	7	29%			6	27%			7	37%			5	31%			0.922
Female child (*n*, %)	7	29%			10	46%			10	53%			6	38%			0.437
**Severe Outcome (Cluster 3)**	**PRS ≤ 15 (*n* = 19)**				**PRS 16–18 (*n* = 32)**				**PRS 19–20 (*n* = 47)**				**PRS ≥ 21 (*n* = 19)**				***p***
	**Mean**	**SE**	**Min**	**Max**	**Mean**	**SE**	**Min**	**Max**	**Mean**	**SE**	**Min**	**Max**	**Mean**	**SE**	**Min**	**Max**	
Child’s age	12	1	6	15	11	1	6	16	11	0	6	16	10	1	6	15	0.392
Age at first clinical diagnosis (months)	22	9	1	148	28	7	0	149	24	6	0	171	19	9	0	170	0.879
Corticoid treatment (months)	41	6	0	94	35	5	0	120	33	4	0	96	36	6	0	74	0.782
No. smokers	2	0	0	5	1	0	0	8	1	0	0	4	1	0	0	6	0.037
BMI (kg/m^2^)	20	1	14	28	19	1	14	29	19	1	14	38	18	1	14	26	0.719
Vitamin A (mg/L)	1	0	0	1	1	0	0	3	1	0	0	1	1	0	0	2	0.197
Vitamin C (mg/L)	7	1	4	12	6	1	3	13	8	1	2	13	6	1	3	9	0.197
Vitamin E (mg/L)	9	1	3	19	11	1	4	21	10	1	4	23	11	1	3	20	0.466
Urban site (*n*, %)	11	58%			17	53%			25	53%			15	79%			0.242
Female child (*n*, %)	7	37%			16	50%			14	30%			8	42%			0.331

**Table 7:** Adjusted odds of asthma outcome per (ln)-unit increase in ambient B[*a*]P concentration, stratified according to biclustering ID and polygenic risk scores.

BIClustering ID	Low Polygenic Score (<4)			High Polygenic Score (≥4)		Overall	
		aOR (95% CI)	*p*	aOR (95% CI)	*p*	aOR (95% CI)	*p*
moderate	ln.B[*a*]P	2.6 (0.7, 9.1)	0.146	3.8 (0.5, 31.5)	0.213	2.4 (1.0, 5.4)	0.041
	**Low Polygenic Score (<18)**			**High Polygenic Score (≥18)**		**Overall**	
		**aOR (95% CI)**	***p***	**aOR (95% CI)**	***p***	**aOR (95% CI)**	***p***
severe outcome	ln.B[*a*]P	2.0 (0.3, 15.8)	0.506	5.2 (0.8, 36.1)	0.092	2.7 (0.8, 9.3)	0.123
